# Supplementary material for: Trends, wealth inequalities and the role of the private sector in caesarean section in the Middle East and North Africa: A repeat cross-sectional analysis of population-based surveys
Source: PLoS One. 2021 Nov 16;16(11):e0259791. doi: 10.1371/journal.pone.0259791 (PMC8594794; doi:10.1371/journal.pone.0259791)
Supplement: S1 Table — (DOCX) [file pone.0259791.s001.docx]

S1 Table. Sample selection

| **Country** | **MICS** | **DHS** | **Study**  **period** | **Original Sample size** | **No consent/ Not completed** | **Never had a child** | **No births in the last 2 years** | **Eligible women** | **Excluded:**  **delivery mode missing** | **Final study sample** |
| --- | --- | --- | --- | --- | --- | --- | --- | --- | --- | --- |
| **Iraq** | 2011 |  | 2009-2011 | 56,445 | 1,251 | 4,079 | 37,121 | 13,994 | 8 | 13,986 |
|  | 2018 |  | 2016-2018 | 31,060 | 400 | 2,254 | 22,156 | 6,250 | 2 | 6,248 |
| **Palestine** | 2010 |  | 2008-2010 | 12,322 | 343 | 845 | 6,805 | 4,329 | 2 | 4,327 |
|  | 2014 |  | 2011-2014 | 13,964 | 597 | 732 | 9,744 | 2,891 | 5 | 2,886 |
| **Tunisia** | 2012 |  | 2009-2012 | 10,514 | 299 | 541 | 8,539 | 1,135 | 1 | 1,134 |
|  | 2018 |  | 2016-2018 | 11,017 | 458 | 621 | 8,726 | 1,212 | 1 | 1,211 |
| **Qatar** | 2012 |  | 2010-2012 | 5,809 | 110 | 2734 | 2,195 | 770 | 3 | 767 |
| **Algeria** | 2013 |  | 2010-2013 | 41,184 | 2,637 | 2,706 | 29,863 | 5,978 | 111 | 5,867 |
| **Sudan** | 2010 |  | 2008-2010 | 18,614 | 1,440 | 6,334 | 5,213 | 5,627 | 32 | 5,595 |
|  | 2014 |  | 2012-2014 | 20,327 | 2,025 | 6,601 | 6,024 | 5,677 | 60 | 5,617 |
| **Egypt** |  | 2008 | 2006-2008 | 16,527 | 0 | 1,749 | 10,243 | 4,535 | 3 | 4,532 |
|  |  | 2014 | 2012-2014 | 21,762 | 0 | 1992 | 13,354 | 6,416 | 0 | 6416 |
| **Jordan** |  | 2012 | 2010-2012 | 11,352 | 0 | 1,048 | 6,626 | 3,678 | 58 | 3620 |
|  |  | 2018 | 2016-2018 | 14,689 | 0 | 1,774 | 9,006 | 3,909 | 145 | 3,764 |
| **Yemen** |  | 2013 | 2011-2013 | 16,656 | 0 | 1,968 | 8,515 | 6,173 | 11 | 6,162 |
