# Supplementary material for: Trends, wealth inequalities and the role of the private sector in caesarean section in the Middle East and North Africa: A repeat cross-sectional analysis of population-based surveys
Source: PLoS One. 2021 Nov 16;16(11):e0259791. doi: 10.1371/journal.pone.0259791 (PMC8594794; doi:10.1371/journal.pone.0259791)
Supplement: S2 Table — Values are percentages (95% Confidence intervals). (DOCX) [file pone.0259791.s002.docx]

S2 Table. The change in the proportion of caesarean section over time.

| **Country** | **Period 1 (2008-2012)** | **Period 2 (2013-2018)** | **Average yearly change** | **Absolute risk difference (95% CI)** |
| --- | --- | --- | --- | --- |
| **Egypt** | 30.6 (29.0; 32.4) | 57.3 (55.6; 59.1) | 4.45% | 26.7 (24.1-29.3) |
| **Tunisia** | 26.7 (23.8; 29.8) | 43.6 (40.5; 46.8) | 2.82% | 16.9 (12.6-21.3) |
| **Iraq** | 22.2 (21.1; 23.3) | 33.2 (31.1; 35.4) | 1.59% | 11.0 (8.6-13.4) |
| **Jordan** | 30.5 (27.7; 33.5) | 28.2 (26.0; 30.6) | -0.38% | -2.3 (-6.0-1.4) |
| **Palestine** | 16.8 (15.6; 18.0) | 20.4 (18.9; 21.9) | 0.9% | 3.6 (1.6-5.6) |
| **Sudan** | 6.8 (5.9; 7.8) | 9.2 (8.1; 10.5) | 0.6% | 2.4 (0.9-4.0) |
| **Qatar** |  | 19.6 (16.1; 23.7) | -- | -- |
| **Algeria** |  | 16.2 (14.9; 17.7) | -- | -- |
| **Yemen** |  | 5.7 (4.9; 6.6) | -- | -- |

Values are percentages (95% Confidence intervals).
